# Die Versorgung von Menschen mit psychischen Erkrankungen in Deutschland aus Perspektive des Gesundheits- und Sozialsystems: Aktuelle Entwicklungsbedarfe

**DOI:** 10.1007/s00103-023-03671-x

**Published:** 2023-03-03

**Authors:** Anke Bramesfeld

**Affiliations:** Institut für Epidemiologie Sozialmedizin und Gesundheitssystemforschung OE 5410, Medizinische Hochschule Hannover, Carl-Neuberg-Str. 1, 30625 Hannover, Deutschland

**Keywords:** Gesundheitssystem, Psychiatrische Versorgung, Langzeitpatienten, Ambulante Versorgung, Personalmangel, Health care system, Psychiatric care, Long-term patients, Outpatient care, Lack of personnel

## Abstract

**Thema:**

Der Artikel gibt einen Überblick über den gegenwärtigen Stand der Versorgung von Menschen mit psychischen Erkrankungen, finanziert durch das Krankenkassen-, das Rehabilitations- und Teilhabesystem und durch die Länder. In den letzten 20 Jahren sind die Versorgungskapazitäten stetig angewachsen. 3 Bereiche mit Entwicklungsbedarfen werden näher beleuchtet: die Koordination der Versorgung für Menschen mit psychischen Erkrankungen und komplexen Hilfebedarfen, die Langzeitunterbringung von Menschen mit schweren psychischen Erkrankungen und herausforderndem Verhalten und ein zunehmender Fachkräftemangel.

**Fazit:**

Deutschland verfügt über ein in weiten Teilen gut bis sehr gut ausgebautes psychiatrisches Versorgungssystem. Bestimmte Gruppen profitieren trotz alledem nicht von den vorhandenen Hilfen, sie finden sich unter anderem als LangzeitpatientInnen in psychiatrischen Kliniken. Modelle für eine koordinierte und ambulant orientierte Versorgung für Menschen mit schweren psychischen Erkrankungen existieren nur vereinzelt. Es mangelt insbesondere an (intensiv) aufsuchender Komplexversorgung und an sozialgesetzbuchübergreifenden Konzepten. Der Fachkräftemangel, der das gesamte Versorgungssystem für Menschen mit psychischen Erkrankungen betrifft, verlangt einen Strukturwandel hin zu stärkerer Ambulantisierung. Erste Instrumente hierfür existieren im krankenkassenfinanzierten System. Sie sollten genutzt werden.

## Abstract (en)

**Subject:**

The article provides an overview on the current state of service provision for people with psychiatric illnesses, financed by health-insurance companies, the rehabilitation and participatory systems, and the German Federal States. Over the last 20 years, service capacities have continually improved. Three areas that are in need of further development are highlighted here: the coordination of services for mentally ill persons with complex needs; the long-term placement of persons with severe mental illness and challenging behavior; and a growing shortage of specialists.

**Conclusions:**

The mental health system in Germany is largely well to very well developed. Despite this, specific groups do not benefit from the available assistance, and these often end up as “long-term patients” in psychiatric clinics. Models for coordinated and outpatient-oriented service provision for persons with severe mental illness exist, but are only sporadic. Intensive and complex outreach services in particular are lacking, as are service concepts that are able to cross the boundaries of social security responsibilities. The shortage of specialists, which affects the whole mental health system, requires restructuring toward a more outpatient-care focus. The first tools for this exist within the health insurance-financed system. They should be used.

## Hintergrund

**Figure 1:**
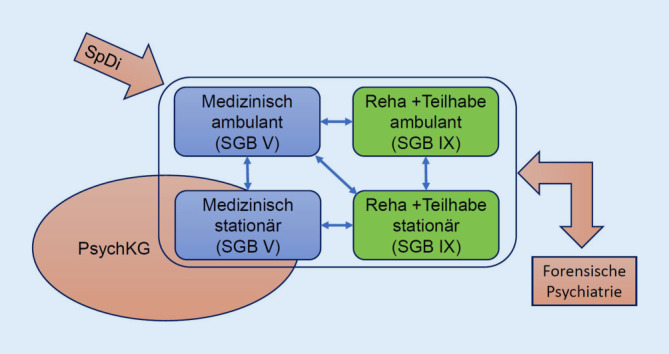

Die Versorgung von Menschen mit psychischen Störungen und Erkrankungen steht im Fokus der gesundheitspolitischen Diskussion sowohl im Kontext der Belastungen durch die Coronapandemie als auch angesichts seit Jahren steigender Inanspruchnahme von Leistungen unter der Diagnose einer psychischen Erkrankung [1]. Die Versorgung erfolgt im stationären und ambulanten Sektor, in der Zuständigkeit von mehreren Sozialgesetzbüchern (SGB), von Ländern und Kommunen sowie durch eine Vielzahl von Leistungserbringern (Abb. 1).

Dem Versorgungssystem für Menschen mit psychischen Erkrankungen ist ein System der Beratung für Menschen mit psychosozialen Bedarfen vorgeschaltet. Dazu gehören u. a. Einrichtungen der Erziehungs‑, Ehe‑, Familien- und Lebensberatung sowie der Teilhabeberatung, aber auch die Suchtberatung und die Beratung durch die Sozialpsychiatrischen Dienste (SpDi). Diese Beratungsangebote sind kommunal organisiert und werden aus Steuermitteln oder auch privat finanziert [2]. Die inhaltliche Grenze von Beratung zur psychiatrischen Versorgung ist nicht immer scharf zu ziehen. Während aber alle Menschen, unabhängig davon, ob bei ihnen eine psychische Erkrankung diagnostiziert wurde, Zugang zum Beratungssystem haben, haben nur Menschen mit einer psychischen Erkrankung, die durch eine ärztliche Diagnose bestätigt wurde, Zugang zum Regelversorgungssystem für Menschen mit psychischen Erkrankungen. Um dieses System soll es im Folgenden gehen.

Die Versorgung für Menschen mit psychischen Erkrankungen gliedert sich in die medizinische (SGB V), die rehabilitative und die Teilhabeversorgung (SGB IX), die als Regelleistungen erbracht werden. Für ältere Menschen mit psychischen Erkrankungen werden bei Pflegebedürftigkeit kompensatorische Leistungen der Pflegeversicherung erbracht. Bei Demenzerkrankungen erfolgt dies nahezu regelhaft. Darüber hinaus erbringen die Länder und Kommunen Leistungen für Menschen mit einer psychischen Erkrankung, wenn diese aus eigener Kraft nicht den Zugang zum Regelversorgungssystem finden oder wenn sie aufgrund einer Straftat aus dem SGB-Regelversorgungssystem herausgefallen sind.

Der folgende Artikel soll eine Übersicht über den gegenwärtigen Stand der Versorgung für Menschen mit psychischen Erkrankungen aus der Perspektive des Gesundheits- und Sozialsystems und unter Berücksichtigung der Versorgungsstrukturen in den genannten Bereichen geben und Entwicklungspotenziale beschreiben.

## Versorgung nach SGB V

Grundlage der Versorgung von Menschen mit psychischen Erkrankungen ist das nach SGB V finanzierte Gesundheitssystem. Hier wird die Diagnose einer psychischen Erkrankung gestellt, die Voraussetzung für den Zugang zu den psychiatriespezifischen Leistungen des Regelversorgungssystems ist.

*Die ambulante Versorgung* bildet die Basis der Versorgung von Menschen mit psychischen Erkrankungen. Die Mehrzahl der Menschen mit psychischen Erkrankungen, insbesondere jene mit weitverbreiteten Erkrankungen wie Depressionen, Angst‑, Demenz- oder Suchterkrankungen werden, wie alle Menschen, die an häufigen Erkrankungen leiden, primär hausärztlich versorgt [3]. Fachspezifisch stehen niedergelassene ÄrztInnen für Psychiatrie und Psychotherapie sowie Kinder- und Jugendpsychiatrie und -psychotherapie mehr oder minder flächendeckend zur Verfügung. Die psychotherapeutische Versorgung erfolgt sowohl durch ÄrztInnen für Psychosomatik als auch ärztliche und psychologische PsychotherapeutInnen. PatientInnen können die fachärztliche und die psychotherapeutische Versorgung ohne Überweisung durch eine andere ärztliche Stelle aufsuchen. Von besonderer Relevanz für Menschen mit schweren psychischen Erkrankungen sind ambulante spezialtherapeutische Interventionen wie häusliche psychiatrische Krankenpflege, Ergo- oder Soziotherapie [4]. Diese bedürfen einer ärztlichen Verordnung.

In den letzten 10 Jahren (2012 bis 2021) hat sich die Anzahl der an der ambulanten Versorgung teilnehmenden ÄrztInnen und PsychotherapeutInnen erhöht: Die Anzahl der ÄrztInnen der Kinder- und Jugendpsychiatrie und -psychotherapie stieg um 15,3 %, die der NervenärztInnen um 11,4 %, ärztlichen PsychotherapeutInnen um 11,2 % und psychologischen PsychotherapeutInnen um 65,7 %. Im Jahr 2021 nahmen rund 37.500 ärztliche und psychologische PsychotherapeutInnen an der vertragsärztlichen Versorgung teil. Allerdings besetzen diese nur rund 25.000 Vertragssitze (Bedarfsplanungsgewichte). 2009 war die Anzahl der PsychotherapeutInnen noch annährend gleich mit der Anzahl der Vertragssitze. Das heißt, es sind zwar sehr viel mehr PsychotherapeutInnen als vor 10 Jahren tätig, diese sind aber vermehrt in Teilzeit als TherapeutIn tätig. Insgesamt ist die Anzahl der Vertragssitze für Psychotherapie im Vergleich zum Zuwachs an TherapeutInnen von 2012 bis 2021 eher moderat um 14 % angestiegen [5].

*Der stationären, stationsäquivalenten und teilstationären Versorgung* bedarf es, wenn die ambulante Versorgung nicht mehr ausreicht. In Deutschland gibt es rund 400 Kliniken für Psychiatrie und Psychotherapie, 260 Kliniken für psychotherapeutische Medizin/Psychosomatik und rund 150 Kliniken für Kinder- und Jugendpsychiatrie und -psychotherapie [6]. Die psychiatrischen Planbetten haben sich seit dem Jahr 2000 um 20 % auf 76.000 im Jahr 2020 erhöht. Dabei betrug der Aufwuchs in der Allgemeinpsychiatrie rund 1500 Betten, in der Kinder- und Jugendpsychiatrie 2000 und in der Psychosomatik rund 9000 Betten [6]. Die stationsäquivalente Leistung (StäB) nach § 115d SGB V ist eine durch Klinken erbrachte aufsuchende Form der Versorgung: Menschen mit der Indikation für eine vollstationäre Behandlung werden von einem multidisziplinären Team der Klinik zu Hause aufgesucht. Seit 2018 wurde die StäB schwerpunktmäßig in Kliniken in Baden-Württemberg, Hessen, Berlin und Umland sowie vereinzelten weiteren Standorten implementiert. 2020 boten 36 Kliniken an 38 Standorten StäB an. StäB wird auch an einigen wenigen Standorten für Kinder und Jugendliche angeboten. 2020 behandelten 3 Viertel der Kliniken, die StäB anboten, weniger als 100 Fälle pro Jahr [7]. Auch wenn mittlerweile mehr Kliniken StäB anbieten, so ist diese Form der Behandlung weiterhin eine Ausnahmeerscheinung.

Tageskliniken sind ein wichtiges Bindeglied zwischen der ambulanten und der vollstationären Versorgung. Insbesondere eigenständige und akut aufnehmende Tageskliniken in großen und ländlichen Regionen mit langen Fahrtwegen zur psychiatrischen Klinik sind von erheblicher Bedeutung für die wohnortnahe Versorgung. Nach Daten der Arbeitsgruppe Psychiatrie der Arbeitsgemeinschaft der obersten Landesgesundheitsbehörden (AOLG), die zurzeit nur bis 2015 vorliegen, nahm die Anzahl der Tagesklinikplätze in den Jahren 2000 bis 2015 um 70 % auf rund 14.000 Plätze zu [8].

Mit Psychiatrischen Institutsambulanzen (PIA), die an jedem Standort einer voll- oder teilstationären psychiatrischen Klinik implementiert werden können, erbringen die psychiatrischen Krankenhäuser auch maßgebliche ambulante Leistungen. Zielgruppe sind Menschen mit schweren und komplexen psychischen Erkrankungen. Gerade in Regionen, die nervenärztlich unterversorgt sind, leisten die PIA damit einen wichtigen Beitrag zur Versorgung der Bevölkerung. PIA arbeiten typischerweise multidisziplinär und haben auch das Potenzial aufsuchend tätig zu sein. Tatsächlich gelingt dies besser in Ländern, in denen die Leistungen der PIA nach einem Leistungskatalog vergütet werden (Bayern, Sachsen), als in solchen, in denen nach Quartalspauschalen abgerechnet wird [9].

In den Modellvorhaben nach § 64b SGB V kann die Behandlung in den Versorgungssettings ambulant (aufsuchend), teil- und vollstationär patientenorientiert und flexibel eingesetzt werden. Dies ist möglich durch eine Vergütung mittels Globalbudgets. Bundesweit sind diese Modellvorhaben eine Ausnahmeerscheinung, angestrebt war mit Inkrafttreten des Gesetzes im Jahr 2013 mindestens 2 Modellvorhaben pro Bundesland zu implementieren. Tatsächlich ist dies nur in rund der Hälfte der Bundesländer gelungen.

## Versorgung nach SGB IX

Menschen mit einer diagnostizierten psychischen Erkrankung, bei denen eine seelische Behinderung droht oder vorliegt – im Sinne einer Beeinträchtigung der Alltagsfunktionen durch die Erkrankung von mehr als 6 Monaten –, haben Anspruch auf Hilfen zur Rehabilitation und Teilhabe. Das SGB IX fasst diese Ansprüche zusammen (Abb. 1). Maßgebliche Bereiche sind dabei:

*Die medizinische Rehabilitation* kann sowohl ambulant als auch stationär erbracht werden. In den Jahren 2000 bis 2017 – das letztverfügbare Jahr, für das vollständige Daten aller Bundesländer außer den Stadtstaaten vorliegen – hat es einen steten Aufwuchs an Betten in psychiatrischen und psychotherapeutisch-psychosomatischen Rehabilitationskliniken gegeben, von insgesamt 25.500 auf 32.000 Betten. Im Jahr 2017 entsprachen damit die stationären Bettenkapazitäten in der psychiatrischen und psychosomatisch-psychotherapeutischen Rehabilitation 40 % der Kapazitäten in Akutkliniken [6].

Ambulante medizinische Rehabilitation für Menschen mit schweren psychischen Erkrankungen findet überwiegend als Komplexleistung mit einer beruflichen Rehabilitation, der Rehabilitation für psychisch Kranke (RPK), statt. Die RPK ist eine Maßnahme zur Bewältigung der Krankheitsfolgen und zur (Wieder‑)Eingliederung in den Arbeitsmarkt. Die Anzahl der Standorte, die eine RPK anbieten, ist über die Jahre stetig gestiegen von 37 Standorten im Jahr 2005 auf 54 im Jahr 2015 [10]. Nach Angabe der Bundesarbeitsgemeinschaft RPK sind es aktuell 61 Standorte mit insgesamt ca. 2000 Plätzen [10]. Bei der Hälfte der Fälle werden die Rehabilitationsleistungen ambulant erbracht. Insgesamt ist von einer Unterversorgung in der medizinischen Rehabilitation bei schweren psychischen Erkrankungen auszugehen. Eine stationäre Rehabilitation kommt hier meist nicht infrage, ambulante oder gar mobile Angebote sind fast nicht vorhanden. Vorrangig mobile Angebote werden bisher in keiner Region regelhaft vorgehalten [11].

*Leistungen zur sozialen Teilhabe* erfolgen durch Assistenzleistungen zum Wohnen und Hilfen in Tagesförderstätten. Assistenzleistungen zum Wohnen werden entweder in besonderen Wohnformen (Heimen) oder in der eigenen Häuslichkeit (ambulant betreutes Wohnen) erbracht. Grundlage ist hier die neu dem SGB IX zugeordnete Eingliederungshilfe. Voraussetzung ist unter anderem das Vorliegen einer seelischen Behinderung. 3 Viertel aller Menschen mit einer seelischen Behinderung, die Assistenzleistungen zum Wohnen erhalten, erhielten diese 2020 in der eigenen Häuslichkeit. Je nach Bundesland, bzw. in Nordrhein-Westfalen die Landschaftsverbände, schwankt diese Quote zwischen 50 % in Sachsen und 80 % in Hamburg bzw. beim Landschaftsverband Rheinland [12].

Nach Daten der AOLG haben sich die Plätze im ambulant betreuten Wohnen von rund 27.000 Plätzen im Jahr 2000 auf 90.000 im Jahr 2015 verdreifacht [8]. Die Kennzahlen der Bundesarbeitsgemeinschaft der überörtlichen Träger der Sozial- und Eingliederungshilfe (BAGüS) weisen auf einen weiteren Aufwuchs der Kapazitäten auch in den letzten Jahren hin: Die Zahl der Leistungsberechtigten mit seelischer Behinderung im ambulant betreuten Wohnen erhöhte sich von rund 128.000 im Jahr 2015 auf 170.000 im Jahr 2020 [12]. Bei Kindern und Jugendlichen mit psychischen Beeinträchtigungen werden diese Leistungen regelhaft durch die Jugendhilfe nach dem SGB VIII erbracht.

Auch die Kapazitäten in besonderen Wohnformen – ehemals Wohnheimen für Menschen mit seelischer Behinderung – haben sich von 37.000 Plätzen im Jahr 2000 auf rund 60.000 im Jahr 2015 erhöht [8]. Es kann angenommen werden, dass sich dieser Trend auch in den letzten 5 Jahren fortgesetzt hat. Die Anzahl der Leistungsberechtigten mit seelischer Behinderung hat sich von 53.000 im Jahr 2015 auf 56.000 im Jahr 2020 erhöht [12]. Es wird geschätzt (eine offizielle Statistik darüber gibt es nicht), dass 10 % der Plätze in Wohnheimen geschlossen geführt werden [13]. Auch die Anzahl der Leitungsberechtigten mit seelischer Behinderung in Tagesförderstätten steigt bundesweit stetig an, seit 2011 um jährlich durchschnittlich 3,8 % [12].

*Leistungen zur Teilhabe am Arbeitsleben* werden mit der Zielsetzung einer Eingliederung in den ersten Arbeitsmarkt über die RPK hinaus in Berufsförderungswerken (28 bundesweit; [14]), in Beruflichen Trainingszentren (23 bundesweit) und in besonderen Angeboten von Bildungsträgern vorgehalten [15]. Auch in den Werkstätten für behinderte Menschen (WfbM) steigt die Bedeutung der seelischen Behinderung stetig: 2011 litten 17 % der in WfbM beschäftigten Menschen an einer seelischen Behinderung, 2020 waren es 20 % [12].

## Versorgung durch die Länder

Alle Länder halten Sozialpsychiatrische Dienste (SpDi) vor. Sie sind kommunal organisiert. Die Kernaufgaben der SpDi umfassen zum einen die niederschwellige Beratung und Betreuung. Als einziger Akteur im Versorgungssystem können SpDi Menschen allein aufgrund von Hinweisen Dritter auf eine möglicherweise vorliegende psychische Erkrankung aufsuchen. Zum anderen intervenieren SpDi bei psychischen Krisen und koordinieren die Versorgung für einzelne Klienten und im Sozialpsychiatrischen bzw. Gemeindepsychiatrischen Verbund [16]. Diese sind mehr oder minder verbindliche Zusammenschlüsse der Leistungserbringer vor Ort. Der Fokus liegt meist auf den Leistungserbringern der Eingliederungshilfe, häufig gemeinsam mit der stationären Versorgung nach SGB V. Angestrebt wird aber eine umfassende Zusammenarbeit sowohl der Leistungserbringer innerhalb der SGBs als auch SGB-übergreifend.

Berlin und Bayern sowie vereinzelte Kommunen anderer Bundesländer haben psychiatrische Krisendienste implementiert. Diese sind in definierten Regionen außerhalb von Geschäftszeiten für Menschen mit psychischen Erkrankungen ansprechbar. Sie arbeiten auch aufsuchend.

Schließlich sind die Länder zuständig für Menschen mit psychischen oder Suchterkrankungen, die unter dem Eindruck dieser Erkrankungen schwerwiegende Straftaten begangen haben, von denen weiter eine Gefahr ausgeht und über die eine Maßregel verhängt wurde. Wie für alle Menschen im Strafvollzug greifen für die Zeit der Verhängung der Maßregel die Sozialgesetzbücher nicht mehr und ein eigenes vom jeweiligen Land getragenes Versorgungssystem der forensischen Psychiatrie übernimmt die Versorgung (Abb. 1).

## Entwicklungsbedarfe der Regelversorgung aus Public-Health-Sicht

**Figure 2:**
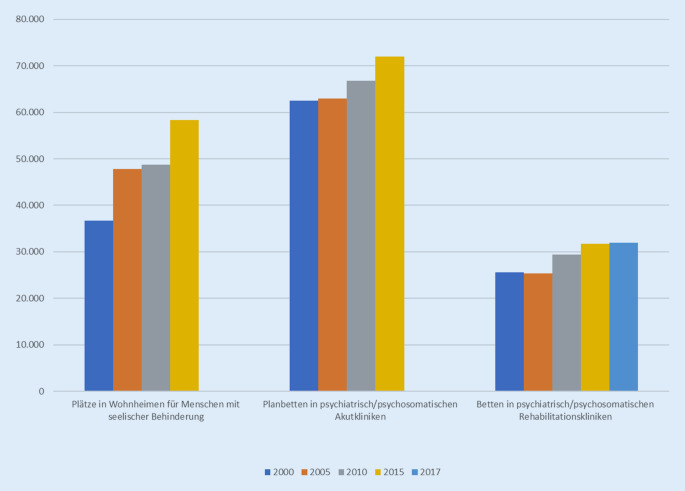

In den Jahren seit der Jahrtausendwende hat es einen kontinuierlichen Aufwuchs sowohl an ambulanten als auch an stationären Versorgungseinrichtungen in fast allen Versorgungsbereichen gegeben, die Menschen mit psychischen Erkrankungen betreffen. Abb. 2 zeigt exemplarisch den Aufwuchs der vollstationären Kapazitäten sowohl beim Wohnen als auch bei der Krankenversorgung in Akut- und Rehabilitationskliniken.

Gleichzeitig haben sich die Kapazitäten für Leistungen zur Teilhabe von Menschen mit seelischer Behinderung erhöht, insbesondere was die ambulanten Assistenzleistungen betrifft. Auch wenn die Genauigkeit der Daten gerade hinsichtlich der Angebote im betreuten Wohnen bzw. der Assistenzleistungen infrage gestellt werden darf, so ist der Trend einer Zunahme der Kapazitäten in allen Versorgungsbereichen unbestritten und wird auch von Daten aus anderen Quellen bestätigt [12]. Auch die Anzahl der Einrichtungen zur beruflichen Rehabilitation ist gestiegen, allerdings hat sich auch der Anteil der Menschen mit einer seelischen Behinderung in einer WfbM für Erwerbsgeminderte erhöht. Dies spricht nicht für eine Verbesserung der gesellschaftlichen Integration von Menschen mit psychischen Erkrankungen.

Trotz des Ausbaus der Versorgung gibt es weiter Herausforderungen für das Versorgungssystem. 3 wesentliche Versorgungsbereiche bzw. -themen sollen im Folgenden differenzierter betrachtet und Entwicklungsbedarfe aufgezeigt werden:

1. die Koordination der Versorgung von Menschen mit psychischen Erkrankungen und komplexen Hilfebedarfen,
2. die Langzeitunterbringung von Menschen mit schweren psychischen Erkrankungen und herausforderndem Verhalten,
3. ein zunehmender Fachkräftemangel, der die Leistungserbringung in fast allen Bereichen der Versorgung beeinflusst.

In der weiteren Diskussion dieser Entwicklungsbedarfe wird auf die Regelversorgung von erwachsenen Menschen fokussiert. Vieles lässt sich auch auf die Versorgung von Kindern- und Jugendlichen oder alten Menschen mit psychischen Erkrankungen übertragen.

## 1. Koordination der Versorgung bei komplexem Hilfebedarf

Menschen mit schweren psychischen Erkrankungen bedürfen vielschichtiger Hilfen, die von den verschiedensten Leistungserbringern innerhalb des Krankenkassen-, des Rehabilitations- und Teilhabesystems erbracht werden [4]. An welchem Punkt einer Krankheitsentwicklung ambulante Versorgung nicht mehr ausreicht, hängt dabei unter anderem von der Ausstattung der ambulanten Versorgung ab. Dass die Koordination der psychiatrischen Hilfen über Sektoren und Leistungsanbieter hinaus zu einer verlässlicheren Versorgung führt und letztendlich Krankenhausaufenthalte vermeiden kann, konnte in zahlreichen Studien nachgewiesen werden [4].

Dieses Wissen versucht die im Dezember 2021 in Kraft getretene „Richtlinie über die koordinierte Versorgung für schwer psychisch Erkrankte“ (KSVPsych-RL) aufzugreifen. Ziel ist die berufsgruppenübergreifende Koordination der ambulanten gesteuerten Versorgung nach SGB V für Menschen mit schweren psychischen Erkrankungen als Regelleistung. Hierfür schließen sich mindestens 10 NervenärztInnen oder PsychotherapeutInnen zu Netzwerkverbünden zusammen und gehen feste Kooperationen mit Leistungserbringern der häuslichen psychiatrischen Krankenpflege, Sozio- oder Ergotherapie und mit psychiatrischen Kliniken ein. In Niedersachsen formieren sich gerade die ersten Netzwerkverbünde.

Die Richtlinie koordiniert vorhandene Regelversorgung, die zum Teil starren Vorgaben im Leistungserbringerrecht überwindet sie nicht. So ist z. B. eine multiprofessionelle, aufsuchende Intensivbehandlung als Komplexleistung nicht Fördergegenstand der KSVPsych-RL. Für eine solche Versorgung gibt es keine gesetzliche Grundlage im SGB V. Sie ist nur begrenzt in PIA möglich, die über einen Leistungskatalog abrechnen (Bayern, Sachsen). Der Koalitionsvertrag 2021–2025 der Bundesregierung sieht aber eine Verbesserung des Zugangs zu ambulanten psychiatrischen Komplexleistungen vor. Die Umsetzung dieses Vorhabens steht noch aus.

Eine weitere Möglichkeit, koordinierte und flexible patientenorientierte Versorgung zu erbringen, sind die Modellprojekte nach § 64b SGB V. Diese aus der Krankenhausversorgung hervorgehenden Modellversuche führen zu einer Verschiebung der Inanspruchnahme von vollstationären Behandlungstagen zugunsten von Behandlung zu Hause und in Tageskliniken. Im Vergleich zur herkömmlichen Krankenhausversorgung erreichen die Modellversuche eine bessere Behandlungskontinuität, verbesserten Zugang zu weiteren Versorgungsangeboten sowie eine Verringerung der Bürokratielast und haben das Potenzial eines geringeren Personalaufwandes [17, 18]. Die Modellvorhaben sind jedoch zeitlich begrenzt und bedürfen separater Vertragsabschlüsse mit jeder einzelnen Krankenkasse, da es sich um keine Regelleistung handelt.

*Zusammenfassend *haben sowohl die ambulant gesteuerten Netzwerke der KSVPsych-RL als auch die von den psychiatrischen Kliniken gesteuerte Versorgung nach § 64b SGB V das inhaltliche Potenzial, Bausteine einer Versorgung zu werden, die komplexe Behandlungsbedarfe besser koordiniert, Kontinuität in der Versorgung stärkt und Alternativen zur klassischen vollstationären Versorgung mit ihren Nebenwirkungen wie Hospitalisierung bietet.

Sowohl KSVPsych-RL als auch Modellvorhaben nach § 64b SGB V sehen eine Kooperation über den SGB-V-Rahmen hinaus – etwa mit den regionalen Sozial- oder Gemeindepsychiatrischen Verbünden – nur als Kann-Bestimmung vor. Eine SGB-übergreifende Kooperation und Koordination von Versorgung ist aber essentiell für das Gelingen der Versorgung von Menschen mit schweren psychischen Erkrankungen.

## 2. Langzeitunterbringung von Menschen mit psychischen Erkrankungen

Mit der Umsetzung der Psychiatrie-Enquete wurden in den 1990er-Jahren die Langzeitbereiche in den psychiatrischen Kliniken aufgelöst, die PatientInnen in Heime oder ambulant betreutes Wohnen umgesiedelt oder gleich ganz entlassen. Fortan war die Lesart, dass es keine LangzeitpatientInnen mehr in psychiatrische Kliniken gäbe. Tatsächlich finden sich heute zumindest PatientInnen in psychiatrischen Kliniken, die dort Monate, teilweise Jahre, auf geschlossenen Akutstationen verweilen. Meist handelt es sich um Menschen mit aggressivem Verhalten, für die keine aufnehmende Einrichtung gefunden werden kann, und/oder Menschen mit einem langfristigen Amtsgerichtsbeschluss zur Unterbringung nach § 1906 Bürgerliches Gesetzbuch (BGB) bei selbstgefährdendem Verhalten. Eine Stichtagserhebung in Niedersachsen ergab, dass Fachkrankenhäuser und psychiatrische Abteilungen gleichermaßen von diesem Phänomen betroffen sind [19]. Psychiatrische Kliniken in ganz Deutschland suchen wohnortnah, wohnortfern und im gesamten Bundesgebiet nach besonderen Wohnformen, die bereit wären diese Menschen aufzunehmen. Eine Erhebung in Mecklenburg-Vorpommern erbrachte, dass rund 40 % der Bewohner von Heimen für Menschen mit seelischer Behinderung außerhalb ihres Heimatlandkreises untergebracht sind [20].

Das Phänomen der LangzeitpatientInnen, die in Akutkliniken leben, steht im Kontrast zu der Entwicklung der Assistenzleistungen zum Wohnen, deren Kapazitäten für Menschen mit seelischer Behinderung in den letzten 20 Jahren stetig angestiegen sind, insbesondere im Bereich des ambulant betreuten Wohnens. Das heißt, die Zunahme an Kapazitäten in den Assistenzleistungen zum Wohnen ist nicht von einer Zunahme an Wohnmöglichkeiten für die am schwersten erkrankten Menschen begleitet. Besorgniserregend ist diese Entwicklung vor dem Hintergrund des weitgehenden Fehlens von Angeboten der medizinischen Rehabilitation für Menschen mit schweren psychischen Erkrankungen, vor allem des Fehlens von ambulanten und mobilen Formen der medizinischen Rehabilitation. Der Entwicklung von Krankheitsverläufen, die in eine abhängige Versorgungsbedürftigkeit beim Wohnen führen, wird so nur wenig entgegengesetzt.

Während also dem Zufluss von Menschen mit psychischen Erkrankungen in das System der Assistenzleistungen aus medizinisch-rehabilitativer Sicht nur wenig entgegengesetzt wird, so scheinen auch die rehabilitative Förderung und Versorgung der Menschen im Assistenzleistungssystem als fraglich ausreichend zu sein, um Selbständigkeit und Krankheitsbewältigung zu fördern und damit wichtige Voraussetzungen für den Ausstieg aus dem System zu schaffen:

- Eine systematische Kooperation von besonderen Wohnformen und Assistenzleistungen mit der psychiatrischen Versorgung nach SGB V ist kein Standard. Wo implementiert, hilft sie aber Krisen von BewohnerInnen im Vorfeld abzufangen und stationären Aufnahmen oder dem Einsatz restriktiver Maßnahmen wie einer geschlossenen Heimunterbringung vorzubeugen [21]. Auch StäB erscheint als ein mögliches Instrument der Krisenbewältigung und Alternative zur vollstationären Aufnahme.
- Neben konzeptionell sehr gut aufgestellten besonderen Wohnformen gibt es auch Hinweise auf solche mit unterentwickelter Umsetzung von Tagesstruktur- und Rehabilitationskonzepten [22].
- Standards in Bezug auf die inhaltlichen Konzepte der besonderen Wohnformen und Assistenzleistungen fehlen, insbesondere auch hinsichtlich des Ziels, BewohnerInnen in eine weniger restriktive Wohnform übersiedeln zu können oder gar aus dem Assistenzsystem zu entlassen.
- Eine systematische indikatorengestützte Qualitätssicherung und -förderung der inhaltlichen Arbeit und der Prozesse in den besonderen Wohnformen und Assistenzleistungen befindet sich erst im Aufbau. Das SGB IX, auch Bundesteilhabegesetz (BTHG) genannt, versucht dort Impulse zu setzen.

*Zusammenfassend* liegt eine oft nicht bedarfsorientierte und integrierte Versorgung vor mit der Folge, dass Menschen mit schwerer psychischer Erkrankung in einem für ihre Bedürfnisse ungeeigneten Setting leben müssen, das Unterstützung bei der sozialen und beruflichen Teilhabe und Bewältigung der Krankheitsfolgen nicht im gewünschten Maß leisten kann [13, 20]. Es bedarf eines Ausbaus von Leistungen zur ambulanten und aufsuchenden Rehabilitation, die der Chronifizierung von Erkrankung vorbeugt und einen Erhalt der Eigenständigkeit auch bei schweren Erkrankungen konsequent fördert.

## 3. Fachkräftemangel

Ein zunehmender alle Professionen betreffender Fachkräftemangel zieht sich als Querschnittsthema durch alle Bereiche der Versorgung von Menschen mit psychischen Erkrankungen. Er steht im Kontext eines Fachkräftemangels im gesamten Bereich der Behandlung und Rehabilitation. Die Versorgung von Menschen mit psychischen Erkrankungen, die sowohl personalintensiv als auch nach wie vor mit einem gewissen Stigma behaftet ist [23], leidet vor allem darunter. So gaben 80 % der psychiatrischen Kliniken im Jahr 2019 an, Probleme bei der Besetzung ärztlicher Stellen zu haben, 73 % hatten Probleme, pflegerische Stellen zu besetzen [24]. Im Mittel waren pro Klinik 10 Pflegestellen unbesetzt. Längst werben psychiatrische Kliniken systematisch ärztliches und pflegerisches Personal aus dem außereuropäischen Ausland an. Kliniken schließen Stationen oder bauen Kapazitäten trotz Bewilligung nicht aus, weil das Personal fehlt. Aber auch im außerklinischen Bereich hinterlässt der Fachkräftemangel Spuren: Nervenarztsitze insbesondere im ländlichen Bereich bleiben unbesetzt, von 34 Sozialpsychiatrischen Diensten in Niedersachsen werden 9 nicht mehr fachärztlich geführt, obwohl dies gesetzlich so vorgesehen ist [25].

Jenseits des demografischen Wandels ist dieser Fachkräftemangel auch im Zusammenhang mit einer Differenzierung und Spezialisierung in der Gesundheitsversorgung zu sehen, die zu einem insgesamt höheren Personalbedarf führt. Wurde im Jahr 1990 bei rund 238.000 berufstätigen ÄrztInnen noch eine „Ärzteschwemme“ beklagt, so herrscht 2021 bei 416.000 berufstätigen ÄrztInnen ein „Ärztemangel“ [26].

Die Steigerung der Attraktivität des Pflegeberufs, das Anwerben von Fachkräften aus dem Ausland und innovative Therapiekonzepte sind sinnvolle Strategien, den Fachkräftemangel kurzfristig zu beheben; mittel- und langfristig wird das Versorgungssystem von Menschen mit psychischen Erkrankungen aber nicht um einen Strukturwandel herumkommen. Dieser Strukturwandel kündigt sich im SGB V bereits mit der Implementierung einer verbindlichen Mindestpersonalausstattung in den psychiatrischen Kliniken durch die Richtlinie zur Personalausstattung in Psychiatrie und Psychosomatik (PPP-RL) an, die hier wie ein Katalysator wirkt, denn bei Nichterfüllung der Personalvorgaben drohen finanzielle Abschläge. Bereits jetzt gibt es Berichte aus Ländern, dass privat geführte Kliniken ihren Versorgungsauftrag kündigen. Wird der durch die Fachkräftesituation getriebene Strukturwandel dem freien Spiel des Marktes überlassen, besteht die Gefahr, dass dies nicht zu einer Verbesserung der Versorgung von Menschen mit schweren psychischen Erkrankungen führen wird.

Ein gesteuerter Strukturwandel im Sinne einer verbesserten Versorgung für Menschen mit psychischen Erkrankungen und eines effizienteren Einsatzes der vorhandenen Fachkräfte sollte sowohl Doppelstrukturen abbauen, ambulante Potenziale stärken und Versorgung besser koordinieren. Doppeluntersuchungen, Parallelbehandlungen oder unkoordinierte Leistungen durch verschiedene Behandelnde müssen vermieden werden. Folgende konkrete Maßnahmen sind unter anderem denkbar:

- Eine konsequente Ambulantisierung der psychiatrischen Versorgung: Damit ambulante Versorgung auch Menschen mit einer schweren psychischen Erkrankung ausreichend Unterstützung bieten kann, bedarf es hierfür eines Ausbaus von StäB, einer besseren Koordination und regionalen Steuerung, insbesondere durch Gemeindespsychiatrische Verbünde und der KSVPsych-RL und einer aufsuchenden ambulanten Intensivbehandlung als Komplexleistung.
- Die klinikbasierten Modellvorhaben nach § 64b SGB V sollten als Regelleistung möglich sein. Sie haben viel Potenzial nicht nur für eine effektivere, sondern auch für eine effizientere Versorgung.
- Enge und regelhafte Kooperationen zwischen Einrichtungen der Assistenzleistungen zum Wohnen und der psychiatrischen Versorgung nach SGB V sollten etabliert werden.

*Zusammenfassend* verfügt das SGB-V-gesteuerte Gesundheitssystem über erste Instrumente für einen Strukturwandel in der Versorgung von Menschen mit psychischen Erkrankungen hin zu einem ambulanteren, koordinierten und effizienteren System. Es bedarf allerdings der flächendeckenden Implementierung der Instrumente und ihrer Implementierung in die Regelversorgung, damit sie ihre Wirkung auch auf Systemebene entfalten können.

## Fazit

Deutschland verfügt über ein in weiten Teilen gut bis sehr gut ausgebautes Versorgungssystem für Menschen mit psychischen Erkrankungen. Dieses wurde in den letzten Jahren stetig erweitert, insbesondere in der psychotherapeutisch-psychosomatischen und der stationär rehabilitativen Versorgung sowie den Assistenzleistungen zum Wohnen und den arbeitsrehabilitativen Angeboten. Trotz alledem gibt es nach wie vor Menschen, die von den Hilfen des psychiatrischen Versorgungssystems nicht profitieren können und sich unter anderem als LangzeitpatientInnen in den psychiatrischen Kliniken wiederfinden. Modelle für eine koordinierte und ambulant orientierte Versorgung auch für Menschen mit schweren psychischen Erkrankungen existieren, sind aber meist nur lokal begrenzt implementiert. Es mangelt insbesondere an (intensiv) aufsuchender Versorgung und an SGB-übergreifenden Konzepten.

Der Fachkräftemangel, der das gesamte Versorgungssystem für Menschen mit psychischen Erkrankungen betrifft, verlangt einen Strukturwandel. Doppelstrukturen sollten abgebaut und die Versorgung auch für Menschen mit komplexen Erkrankungen weiter ambulantisiert werden. Als Instrumente in der SGB-V-gesteuerten Versorgung existieren hierfür Modelle sowohl für den Krankenhausbereich als auch für die ambulante kassenärztlichen Versorgung. Die Instrumente müssen aber auch implementiert und weiterentwickelt werden, damit sie ihre Wirkung entfalten können.
